# KNOWLEDGE MOBILIZATION TO CREATE DEMENTIA-INCLUSIVE NEIGHBORHOODS: AN EDUCATIONAL AND AWARENESS-RAISING VIDEO

**DOI:** 10.1093/geroni/igae098.1337

**Published:** 2024-12-31

**Authors:** Cari Randa-Beaulieu, Habib Chaudhury, Kishore Seetharaman, Lillian Hung, Joey Wong, Lily Ren

**Affiliations:** Alzheimer Society of British Columbia, Vancouver, British Columbia, Canada; Simon Fraser University, Vancouver, British Columbia, Canada; Simon Fraser University, Vancouver, British Columbia, Canada; University of British Columbia, Vancouver, British Columbia, Canada; University of British Columbia, Vancouver, British Columbia, Canada; University of British Columbia, Vancouver, British Columbia, Canada

## Abstract

To promote community engagement and increase awareness of dementia-inclusive neighborhood built environment, we have co-created with people with lived experience (PLWE) multiple knowledge mobilization (KM) resources. These resources include an educational and advocacy video and photo exhibit showcasing the project findings to stakeholders and the general public in British Columbia, Canada and beyond. A seven-minute documentary video was produced to illustrate the lived experiences of three people living with dementia in Metro Vancouver and Prince George. This video is one of several public engagement tools for dialogue and challenging stigma surrounding living with dementia in the community that will be mobilized during this phase of the project. This presentation will share the process of community engagement based on the World Café method in two municipalities in Metro Vancouver, Canada. The World Café method encourages conversations, collaborative learning, and generation of new ideas with the video and photo exhibit. This KM project aims to increase awareness and understanding of the features of a dementia-inclusive neighborhood built environment and advocate for positive actions for a broad range of stakeholders, including advocacy groups, community-based organizations, municipalities, and the general public. In partnership with the City of Burnaby and the City of Richmond in Metro Vancouver, these KM activities maximize the impact of the DemSCAPE study’s findings and model various evidence-based approaches to achieving the vision and objectives for developing dementia-inclusive communities.

